# The Postoperative Lymphocyte to Monocyte Ratio Change Predicts Poor Clinical Outcome in Patients with Esophageal Squamous Cell Carcinoma Undergoing Curative Resection

**DOI:** 10.1155/2020/1451864

**Published:** 2020-04-22

**Authors:** Qian Song, Jun-zhou Wu, Hui-fen Jiang, Sheng Wang, Shu-nv Cai

**Affiliations:** ^1^Department of Clinical Laboratory, Zhejiang Cancer Hospital, Institute of Cancer and Basic Medicine (ICBM), Chinese Academy of Sciences; Department of Clinical Laboratory, Cancer Hospital of the University of Chinese Academy of Sciences, Hangzhou, Zhejiang, China; ^2^Zhejiang Cancer Hospital, Institute of Cancer and Basic Medicine (ICBM), Chinese Academy of Sciences; Cancer Research Institute, Cancer Hospital of the University of Chinese Academy of Sciences; Cancer Research Institute, Hangzhou, Zhejiang, China; ^3^Department of Anesthesiology, Zhejiang Cancer Hospital, Institute of Cancer and Basic Medicine (ICBM), Chinese Academy of Sciences; Department of Anesthesiology, Cancer Hospital of the University of Chinese Academy of Sciences, Hangzhou, Zhejiang, China

## Abstract

**Background:**

Postoperative lymphocyte to monocyte ratio (post-LMR) change (LMRc) reflects the dynamic change of balance between inflammatory reaction and immune reaction after curative operation. An elevated preoperative LMR (pre-LMR) has been shown to be a prognostic factor in patients with esophageal squamous cell carcinoma (ESCC), but the clinical value of the LMRc remains unknown.

**Methods:**

674 patients in ESCC undergoing curative operation were enrolled in this study. LMRc (LMRc = pre‐LMR–post‐LMR) was counted on the basis of data within one week before and after operation. The median of LMRc was chosen to be the optimal cut-off value to evaluate the prognostic value of LMRc.

**Results:**

Kaplan-Meier curves revealed that LMRc ≤ 1.59 was significantly associated with worse overall survival (OS) (*P* = 0.003) and disease-free survival (DFS) (*P* = 0.008). Multivariate analysis suggested that LMRc could serve as an independent prognostic predictor for both OS (*P* = 0.006, HR = 0.687, 95% CI 0.526-0.898) and DFS (*P* = 0.003, HR = 0.640, 95% CI 0.476-0.859).

**Conclusions:**

LMRc is a promising prognostic predictor for predicting the worse clinical outcome in patients with ESCC undergoing curative operation.

## 1. Background

The incidence of esophageal cancer is increasing, with an estimated 572,000 new cases globally in 2018. In China, esophageal carcinoma is the fifth most common carcinoma and the fourth leading cause of carcinoma mortality [1]. Esophageal squamous cell carcinoma (ESCC) accounts for 90% of all cases in China [2, 3]. Despite intensive study aimed at developing therapies, the overall prognosis of patients including those with curative resection remains poor [4, 5]. Further studies are needed to identify new prognostic or predictive biomarkers that could help stratify patients for treatment.

Systemic inflammation plays a striking part in cancer development and progression [6]. Plentiful studies report that inflammatory biomarkers, for example, neutrophil to lymphocyte ratio (NLR), lymphocyte to monocyte ratio (LMR), and platelet to lymphocyte ratio (PLR), could be an independent prognostic factor in various cancers [7–11].

The treatment such as surgery and chemotherapy could cause change; therefore, the change of inflammatory biomarkers has been paid attention to recently. The dynamic change of NLR could be a better prognostic predictor in several cancers including gastric cancer [12], lung cancer [13], and kidney cancer [14]. Change in LMR could predict the efficacy of chemotherapy in advanced non-small-cell lung cancer [15]. The change of PLR was an independent prognostic predictor for clinical outcome in patients with hepatocellular carcinoma [16]. However, whether the dynamic change of systemic inflammatory biomarkers in patients with ESCC is associated with clinical outcome remains unclear. Therefore, the purpose of this study is to explore the relationship between the clinical outcome of ESCC and the dynamic change of systemic inflammatory response, including the change of neutrophil to lymphocyte ratio (NLRc), the change of lymphocyte to monocyte ratio (LMRc), and the change of platelet to lymphocyte ratio (PLRc). The present study is aimed at evaluating whether LMRc has a significant relationship with overall survival (OS) and disease-free survival (DFS) in patients with ESCC.

## 2. Methods

### 2.1. Patient Selection

We retrospectively analyzed the clinical data of consecutive patients with ESCC at the Cancer Hospital of the University of Chinese Academy of Sciences, Hangzhou, China, which had received curative resection between Feb. 2008 and Feb. 2015. The eligibility criteria included histologically confirmed resectable ESCC. Individuals were excluded from the present study if they have received chemotherapy or radiotherapy before the surgery. The laboratory data, including preoperative neutrophil, lymphocyte, monocyte, and blood platelet, was obtained by preoperative examination one week before surgery and postoperative evaluation one week after surgery. As a result, 674 patients with newly diagnosed ESCC were enrolled in the present research. Follow-up was performed until Aug. 2016. Our research was approved by the Ethics Committee of the Cancer Hospital of the University of Chinese Academy of Sciences. Informed consent was obtained from all individuals.

### 2.2. Statistical Analysis

The endpoints of this study were OS, which was calculated from the time of diagnosis to the time of any cause of death and DFS that was between diagnosis and occurrence of local recurrence or distant metastases. NLRc (NLRc = pre‐NLR–post‐NLR), LMRc (LMRc = pre‐LMR–post‐LMR), and PLRc (PLRc = pre‐PLR–post‐PLR) were analyzed as continuous variables, which are expressed as median and interquartile range. All clinical features were counted as categorical variables, which are presented as numbers and percentage. Chi-square tests were used to evaluate the relationship between LMRc and clinical features in patients with ESCC. The median of NLRc, LMRc, and PLRc were chosen to be the optimal cut-off value. OS and DFS were calculated using Kaplan-Meier curves and analyzed by the log-rank test. The Kaplan-Meier curve and the log-rank test were analyzed by GraphPad Prism 7 software. The hazard ratio, 95% confidence interval, and *P* value were estimated using COX regression analyses. Statistical analyses were performed using the SPSS, version 19.0, statistical software. All the statistical analysis was two sided, and *P* < 0.05 was regarded as statistical significance.

## 3. Results

### 3.1. Patient Characteristics

We enrolled 674 patients with ESCC who had received curative resection between Feb. 2008 and Feb. 2015. The median age of all individuals was 61 years (range: 35-81 years). The patients with less than 60 years were 310 (46.0%), and the patients with more than 60 years were 364 (54.0%). 576 (85.5%) cases were male, and 98 (14.5%) cases were female. There were 115 (17.1%) with stage 1a-1b, 226 (33.5%) with stage 2a-2b, and 333 (49.4%) with stage 3a-3c. There were 293 (43.5%) cases without lymph node metastasis, and 381 (56.5%) cases with lymph node metastasis. There were 468 (69.4%) patients without vessel invasion, and 206 (30.6%) patients with vessel invasion. 251 (37.2%) patients have nerve infiltration, while 423 (62.8%) patients have no nerve infiltration.  Table 1 summarizes the clinical features of all patients in ESCC undergoing curative surgical resection, which was, in some cases, followed by adjuvant therapies including chemotherapy and radiotherapy.

### 3.2. Differences in OS and DFS according to LMRc

**Figure 1:**
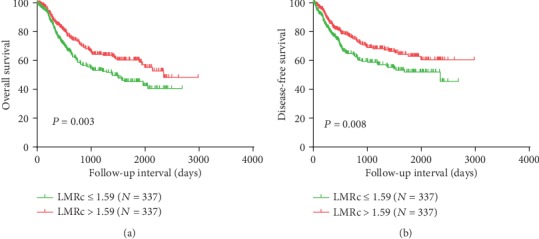
Overall survival and disease-free survival analysis according the LMRc (a, b).

We chose the median as the cut-off value of -3.02 for NLRc, 1.59 for LMRc, and -104.89 for PLRc. We found that patients with lower LMRc (LMRc ≤ 1.59) were more likely to be male (*P* < 0.001), have increased hospital time (≥14 days) (*P* = 0.002) and lower dynamic change for NLR (*P* < 0.001), LMR (*P* < 0.001), and PLR (*P* = 0.003). Lower LMRc (LMRc ≤ 1.59) predicts worse clinical outcomes for OS (*P* = 0.003) and DFS (*P* = 0.008) (Figure 1). Lower NLRc and lower PLRc have no significant relationship with OS and DFS (data not shown). Spearman's analysis showed that LMRc were correlated with sex (*P* < 0.001) and hospital time (*P* = 0.019) (Table 2).

Univariate analysis indicated lower LMRc, pathology grade, depth of tumor, lymph node metastasis, pathological stage, vessel invasive, and nerve infiltration as poor prognostic factors for OS (Table 3). In the multivariate analysis, we identified LMRc (*P* = 0.006), pathology grade (*P* = 0.020), lymph node metastasis (*P* < 0.001), and nerve infiltration (*P* = 0.002) as independent prognostic factors for OS (Table 3).

For DFS, in the multivariate analysis that included LMRc, pathology grade, lymph node metastasis, pathological stage, nerve infiltration, and treatment regimen proven to be significant factors in a univariate analysis, we found that LMRc, pathology grade, lymph node metastasis, nerve infiltration, and treatment regimen could be independent prognostic predictors (for LMRc: HR = 0.640; 95% CI 0.476-0.859; *P* = 0.003; Table 4).

## 4. Discussion

In the present study, we demonstrated for the first time that lower LMRc (LMRc ≤ 1.59) represents a novel independent poor prognostic biomarker in patients with ESCC undergoing curative resection. Accumulating studies have demonstrated that systemic inflammatory response, particularly NLR, LMR, and PLR, could be an independent prognostic predictor in a variety of carcinomas including ESCC [9, 17, 18]. The dynamic change of systemic inflammatory biomarkers reflects the change between the inflammation response and immune response in patients after treatments. Recently, some studies concentrated on the relationship between the dynamic change of systemic response and clinical outcome in patients after therapies. Postoperative elevation of NLR predicts poor clinical outcome in some cancers such as gastric cancer [12], lung cancer [13], and kidney cancer [14]. A retrospective study with 65 patients following esophageal resection proved that a higher NLRc predicts complications [19]. LMRc represents the prognostic factor in patients with lung cancer who received chemotherapy [15]. PLRc was associated with prognosis in patients with hepatocellular carcinoma [16]. To the best of our knowledge, in patients with ESCC, up to now, a potential prognostic value has not been investigated. Therefore, we evaluated the prognostic significance of NLRc, LMRc, and PLRc regarding two different endpoints.

We found that lower LMRc (*LMRc* ≤ 1.59) was significantly associated with gender (male) and hospital time (≥14 days). The incidence and mortality rates in male are 2-fold to 3-fold than those in female globally [1]. The eligibility criteria were consecutive patients with newly diagnosed ESCC who received curative surgical resection. There is a possibility that LMRc is related to gender. The relationship between LMRc and hospital time indicated that lower LMRc might be in a bad condition after surgery. Patients with lower LMRc may have complications, including fever or infection. Therefore, lower LMRc had significant longer hospital time after operation.

To date, research has emerged that shows inconsistent results about the prognostic value of microvascular invasion in patients with ESCC [20].The prognostic value of microvascular invasion in ESCC was investigated in various studies, and many suggested microvascular invasion as a crucial prognostic factor in ESCC and is related to adverse prognosis [21–24], whereas some did not reach any conclusive results indicating that microvascular invasion is correlated to the clinical outcome of ESCC [25, 26]. In the present study, we did not find that microvascular invasion could be an independent prognostic indicator. However, LMRc was an independent prognostic predictor for OS and DFS using multivariate analysis. These findings may contribute to explaining why lower LMRc are associated with hospital time (≥14 days). In our study, we found that NLRc and PLRc had no significant association with OS or DFS. Future studies about different clinical laboratories and races are needed to prove the findings. This study may impact the treatment practice for ESCC.

Although the molecular mechanism behind this prognostic significance remains hypothetical, published research side with our clinical findings. Monocytes, which constitute about 5% of the circulating leukocyte pool, play a crucial part in innate immunity [27]. Tumors are infiltrated with immune competent cells reflecting the antitumor response. Tumor-associated macrophages (TAMs), which are derived from monocytes, are recruited to the microenvironment by chemotactic factors [28]. TAMs play a promising role in angiogenesis, invasion, and worse clinical outcome in various cancers [29–31]. Therefore, the absolute monocyte count may represent formation or presence of TAMs. On the one hand, TAMs promote tumor through stimulating the growth of cancer cells, promoting migration and metastasis [32]. Moreover, TAMs, which produce enzymes and inhibitors digesting the extracellular matrix, contribute to tumor invasion and migration [33, 34]. On the other hand, TAMs suppress the immune response by secreting chemokine that recruit T cell subsets without cytotoxic function [35]. A growing number of clinical research support the protumor role of TAMs in cancers, showing that TAMs could be an independent prognostic predictor in various cancers [36]. To reinforce the prognostic value of monocytes, we combined them with lymphocyte. Lymphocytes, particularly cytotoxic lymphocytes, function as the host defense against cancer cells and are applied in immunotherapy [37, 38]. Lymphocytopenia is a well-known result of a systemic inflammatory response accompanying malignant diseases [37]. The advantage of the present study is the large sample size. However, some shortcomings have to be taken into account, mostly based on the retrospective study and a single-center design.

## 5. Conclusions

To the best of our knowledge, our study is the first report indicating that LMRc is a novel independent prognostic factor in patients with ESCC undergoing curative resection. Large-scale prospective research are needed to prove our findings.

## Figures and Tables

**Table 1:** Demographic and clinical data of 674 ESCC patients according to LMR change.

Characteristics	*N* = 674 (%)	LMRc		*P* value
		≤1.59 (*N* = 337) (%)	>1.59 (*N* = 337) (%)	
Sex				
Male	576 (85.5)	307 (91.1)	269 (79.8)	**<0.001**
Female	98 (14.5)	30 (8.9)	68 (20.2)	
Age				
≤60 years	310 (46.0)	146 (43.3)	164 (48.7)	0.164
>60 years	364 (54.0)	191 (56.7)	173 (51.3)	
Pathology grade				
Well	49 (7.4)	23 (7.0)	26 (7.8)	0.304
Middle	446 (67.5)	231 (70.0)	215 (65.0)	
Poorly	164 (24.8)	76 (23.0)	88 (26.6)	
Undifferentiated	2 (0.3)	0 (0)	2 (0.6)	
Depth of tumor				
T1a–1b	64 (9.5)	29 (8.6)	35 (10.4)	0.670
T2	129 (19.1)	63 (18.7)	66 (19.6)	
T3	481 (71.4)	245 (72.7)	236 (70.0)	
Lymph node metastasis				
N0	293 (43.5)	150 (44.5)	143 (42.4)	0.142
N1	212 (31.5)	101 (30.0)	111 (32.9)	
N2	117 (17.4)	53 (15.7)	64 (19.0)	
N3	52 (7.7)	33 (9.8)	19 (5.6)	
Pathological stage				
1a–1b	115 (17.1)	56 (16.6)	59 (17.5)	0.949
2a–2b	226 (33.5)	113 (33.5)	113 (33.5)	
3a–3c	333 (49.4)	168 (49.9)	165 (49.0)	
Vessel invasive				
Yes	206 (30.6)	101 (30.0)	105 (31.2)	0.738
No	468 (69.4)	236 (70.0)	232 (68.8)	
Nerve infiltration				
Yes	251 (37.2)	125 (37.1)	126 (37.4)	0.936
No	423 (62.8)	212 (62.9)	211 (62.6)	
Treatment regimen				
S	457 (67.8)	224 (66.5)	233 (69.1)	0.757
S+postoperative C	155 (23.0)	81 (24.0)	74 (22.0)	
S+postoperative CRT	62 (9.2)	32 (9.5)	30 (8.9)	
Hospital time				
≤14 days	560 (83.1)	265 (78.6)	295 (87.5)	**0.002**
>14 days	114 (16.9)	72 (21.4)	42 (12.5)	
NLRc				
Median	-3.02 (-4.64–-1.69)	-2.26 (-3.93–-0.95)	-3.78 (-5.39–-2.49)	**<0.001**
LMRc				
Median	1.59 (0.71-2.69)	0.71 (0.05-1.19)	2.68 (2.02-3.54)	**<0.001**
PLRc				
Median	-104.89 (-162.16–-54.85)	-100.65 (-159.19–-38.38)	-108.64 (-163.21–-72.47)	**0.003**

**Table 2:** Spearman's analysis of correlation between LMR change and clinicopathological features.

Variables	LMRc	
	Spearman correlation	*P* value
Sex	-0.198	**<0.001**
Age (years)	-0.056	0.144
Pathology grade	0.019	0.619
Depth of tumor	-0.04	0.3
Lymph node metastasis	-0.009	0.811
Pathological stage	-0.02	0.604
Vessel invasive	0.014	0.725
Nerve infiltration	-0.004	0.91
Treatment regimen	-0.041	0.291
Hospital time (days)	-0.09	**0.019**

**Table 3:** Overall survival analyses according to LMR change in 674 patients with ESCC.

Variables	Univariate			Multivariate		
	HR	95% CI	*P* value	HR	95% CI	*P* value
NLRc	1.013	0.972-1.055	0.542			
LMRc	0.670	0.515-0.870	**0.003**	0.687	0.526-0.898	**0.006**
PLRc	1.000	0.998-1.001	0.485			
Sex	1.249	0.841-1.855	0.270			
Age (years)	1.000	0.983-1.017	0.999			
Pathology grade	1.566	1.228-1.998	**<0.001**	1.357	1.049-1.757	**0.020**
Depth of tumor	1.506	1.173-1.933	**0.001**	1.385	0.994-1.930	0.055
Lymph node metastasis	1.738	1.528-1.978	**<0.001**	1.746	1.416-2.154	**<0.001**
Pathological stage	1.897	1.538-2.342	**<0.001**	0.811	0.555-1.186	0.280
Vessel invasive	1.770	1.356-2.310	**<0.001**	1.172	0.875-1.570	0.287
Nerve infiltration	1.841	1.419-2.390	**<0.001**	1.535	1.164-2.025	**0.002**
Treatment regimen	1.012	0.9-1.137	0.847			
Hospital time (days)	1.007	0.997-1.016	0.169			

**Table 4:** Disease-free survival analyses according to LMR change in 674 patients with ESCC.

Variables	Univariate			Multivariate		
	HR	95% CI	*P* value	HR	95% CI	*P* value
NLRc	1.013	0.976-1.060	0.591			
LMRc	0.677	0.507-0.905	**0.008**	0.640	0.476-0.859	**0.003**
PLRc	1.000	0.998-1.001	0.617			
Sex	1.210	0.788-1.858	0.383			
Age (years)	0.992	0.973-1.010	0.376			
Pathology grade	1.652	1.264-2.157	**<0.001**	1.515	1.140-2.014	**0.004**
Depth of tumor	1.144	0.902-1.450	0.266			
Lymph node metastasis	1.598	1.377-1.853	**<0.001**	1.534	1.222-1.926	**<0.001**
Pathological stage	1.558	1.257-1.932	**<0.001**	0.824	0.595-1.140	0.242
Vessel invasive	1.292	0.949-1.760	0.104			
Nerve infiltration	1.623	1.213-2.170	**0.001**	1.580	1.160-2.150	**0.004**
Treatment regimen	1.385	1.233-1.556	**<0.001**	1.284	1.133-1.45	**<0.001**
Hospital time (days)	1.000	0.989-1.012	0.975			

## Data Availability

The datasets used in the present research are available from the corresponding author on reasonable request.

## Abbreviations

post-LMR: Postoperative lymphocyte to monocyte ratio
LMRc: Postoperative lymphocyte to monocyte ratio change
pre-LMR: Preoperative lymphocyte to monocyte ratio
ESCC: Esophageal squamous cell carcinoma
OS: Overall survival
DFS: Disease-free survival
NLR: Neutrophil to lymphocyte ratio
LMR: Lymphocyte to monocyte ratio
PLR: Platelet to lymphocyte ratio
NLRc: The change of neutrophil to lymphocyte ratio
PLRc: The change of platelet to lymphocyte ratio
HR: Hazard ratio
95% CI: 95% confidence interval
P: Probability.
